# Density of Wild Prey Modulates Lynx Kill Rates on Free-Ranging Domestic Sheep

**DOI:** 10.1371/journal.pone.0079261

**Published:** 2013-11-20

**Authors:** John Odden, Erlend B. Nilsen, John D. C. Linnell

**Affiliations:** Norwegian Institute for Nature Research, Trondheim, Norway; Université de Sherbrooke, Canada

## Abstract

Understanding the factors shaping the dynamics of carnivore–livestock conflicts is vital to facilitate large carnivore conservation in multi-use landscapes. We investigated how the density of their main wild prey, roe deer *Capreolus capreolus*, modulates individual Eurasian lynx *Lynx lynx* kill rates on free-ranging domestic sheep *Ovis aries* across a range of sheep and roe deer densities. Lynx kill rates on free-ranging domestic sheep were collected in south-eastern Norway from 1995 to 2011 along a gradient of different livestock and wild prey densities using VHF and GPS telemetry. We used zero-inflated negative binomial (ZINB) models including lynx sex, sheep density and an index of roe deer density as explanatory variables to model observed kill rates on sheep, and ranked the models based on their AICc values. The model including the effects of lynx sex and sheep density in the zero-inflation model and the effect of lynx sex and roe deer density in the negative binomial part received most support. Irrespective of sheep density and sex, we found the lowest sheep kill rates in areas with high densities of roe deer. As roe deer density decreased, males killed sheep at higher rates, and this pattern held for both high and low sheep densities. Similarly, females killed sheep at higher rates in areas with high densities of sheep and low densities of roe deer. However, when sheep densities were low females rarely killed sheep irrespective of roe deer density. Our quantification of depredation rates can be the first step towards establishing fairer compensation systems based on more accurate and area specific estimation of losses. This study demonstrates how we can use ecological theory to predict where losses of sheep will be greatest, and can be used to identify areas where mitigation measures are most likely to be needed.

## Introduction

Large carnivores live at low densities, and in many parts of the world their conservation is dependent on integrating carnivores into multi-use landscapes [1]. Predation on livestock is one of the major conflicts between humans and large carnivores, and depredation occurs where livestock overlap in distribution with large carnivores [2]. Livestock losses to carnivores are highly variable, both spatially and temporally. Among the factors contributing to this variation are the species and densities of predator and livestock, husbandry practises, size ratio between carnivores and livestock, landscape characteristics, and wild prey densities [2]–[5]. Understanding the dynamics of carnivore–livestock conflicts in such landscapes is vital to effectively achieve large carnivore conservation.

Among the factors that have received attention in previous studies is the effect that distribution and density of wild ungulate prey might have on livestock losses [6]. Previous studies have provided seemingly contradictory results. For example in Eurasian lynx *Lynx lynx* and wolf *Canis lupus* locally high densities of wild prey can increase depredation [7]–[10], whereas other studies of wolves and coyotes have found the opposite [6], [11]–[14]. We propose two models to explain these seemingly contradictory findings. The *attraction model* can explain elevated predation risk for livestock in areas with high densities of wild prey. The model proposes that locally high densities of wild prey will attract carnivores to these patches, and thus also induce elevated risk for livestock. This model assumes that carnivore habitat use is a function of wild prey densities, and that livestock are depredated if they are encountered by chance while the carnivore is searching for wild prey. To explain reduced predation on livestock in areas with high densities of wild prey, the *energetic model* predicts that regionally high densities of wild prey will reduce predation on livestock primarily because there is no need for predators to kill livestock to satisfy their energetic needs. An additional effect comes from the possibility that searching time is reduced at high wild prey density thus reducing the probability of chance encounters with domestic prey. Previous studies supporting the *energetic model* have been conducted by comparing losses in areas of different densities of wild prey [15]–[21] or through scat analysis [6], [12]–[14], [22], [23]. However, such results could also arise from differences in carnivore densities and are not an adequate test of the model.

The difference between the energetic and the attraction models may also simply be a matter of scale. In large areas (home range scales) with low densities of wild prey, carnivores may increase depredation on livestock to compensate for having few prey (thus supporting the *energetic model*), but in areas where wild prey is widely distributed and abundant, carnivores may spend most time in the most prey-rich patches, leading to high encounter rates, and therefore greater incidental depredation on livestock (thus supporting the *attraction model*). We have previously found support for the *attraction model* on a small scale [10]. To rigorously test the *energetic model*, we here use data on lynx kill rates on free-ranging domestic sheep from radio-collared lynx in south-eastern Norway, along a gradient of different livestock and wild prey densities.

## Methods

### Ethic Statement and Sampling

We captured 109 lynx between 1995 and 2011. Adult lynx and juveniles (>5 months) were captured in walk through box-traps, spring-loaded foot-snares, treed using trained dogs, or immobilized from cars and helicopters in a few cases. In addition, kittens were captured by hand in natal lairs. Lynx were immobilized with medetomidine-ketamine, following pre-established protocols [24]–[26]. All capture methods were constantly refined to minimise the risk of injury or death to the animals. In particular, the design and alarms of box traps and snares were modified to allow response time of less than 12 hours (average 5 hours), and 20 minutes, respectively, and a safety net was used when animals were treed with hounds. All capture and handling procedures were approved by the Norwegian Experimental Animal Ethics Committee and followed their ethical requirements for research on wild animals (permit numbers 2012/206992, 2010/161554, 2010/161563, 08/127430, 07/81885, 07/7883, 2004/48647, 201/01/641.5/FHB, 127/03/641.5/fhb, 1460/99/641.5/FBe, 1081/97/641.5/FBe, and NINA 1/95). In addition, permits to capture wild animals were provided by the Norwegian Directorate for Nature Management. Of the 109 lynx, 61 were equipped with VHF radio-collars (Telonics MOD-335 transmitter, Telonics Inc., Mesa, AZ, USA), 15 received free-floating intra-peritoneal implant transmitters (Telonics IMP/150/L and IMP/400/L implantable transmitter with mortality sensor), 7 received store-on-board GPS collars, (2 Lotek 3300SL, Lotek Wireless Inc., Ontario, Canada, and 5 Televilt Posrec 300, Followit AB., Lindesberg, Sweden), and 26 received GPS-GSM collars (4 Tellus 1C, Followit AB and 22 Vectronic GPS PLUS, Vectronic Aerospace GmbH, Berlin, Germany).

### The Socio-economic System

The conflict between carnivores and livestock is relatively high in Norway because of a grazing system based on free-grazing unguarded sheep in carnivore habitat [27], [28]. In Norway, there is a legal requirement that all losses to large carnivores should be fully compensated. An *ex post facto* compensation system [29] is based on an estimation of losses, and on a national level 334,159 sheep and lambs have been compensated as being killed by large carnivores and golden eagles (at a cost of EUR 82 579 801) during the last ten years [30]. Since 1996, an estimated lynx population of between 259 and 486 individuals has been held responsible for killing 6125 to 10 093 sheep annually, corresponding to an annual average of 22 sheep killed per lynx (± SD 2.2) [31]. However, there is a large degree of uncertainty in the magnitude of depredation, because only a small fraction (4–9%) of the compensated sheep is subject to a formal post mortem. The remaining numbers are estimated based on various studies of mortality rates of sheep and lambs [32]–[35].

### Study Areas

**Figure 1:**
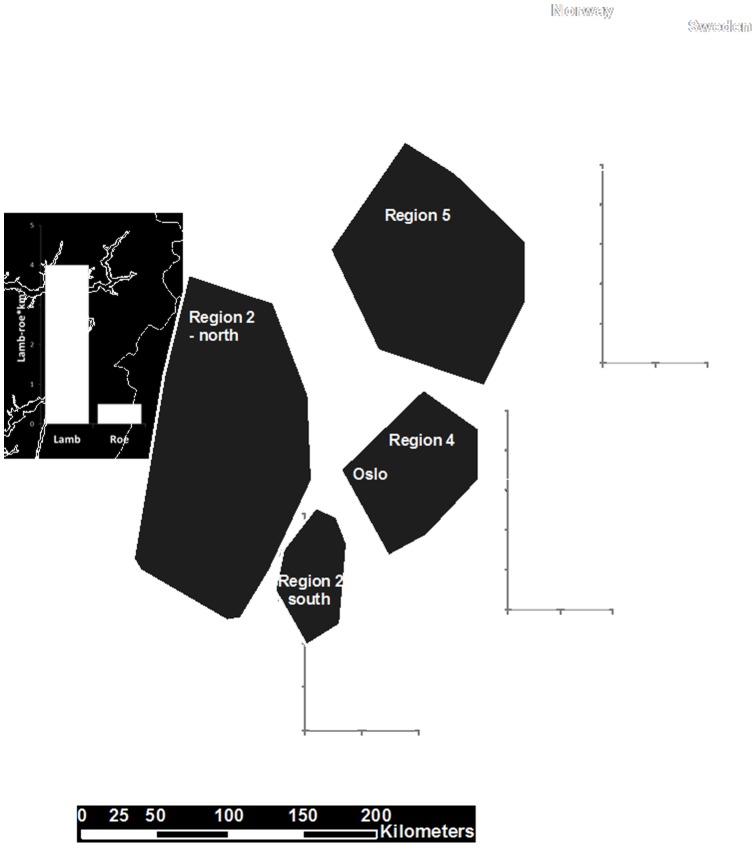
Map of the study area, showing the 4 areas of data collection of lynx (*Lynx lynx*) predation on sheep from 1995 to 2011. The shaded areas indicate the four study areas, Region 5 (Hedmark County 1995–1999), Region 4 (Oslo, Akershus, Østfold Counties 2001–2006), Region 2 north (Buskerud, Telemark, and Oppland Counties 2006–2011), and Region 2 south (Buskerud, Telemark, and Vestfold Counties 2006–2011). For each of the 4 study areas the estimated roe deer and lamb densities are indicated as number of lambs or roe deer per square kilometre.

Data on lynx kill rates on free-ranging domestic sheep were collected in a 57,000 km^2^ study area in south-eastern Norway from 1995 to 2011. The area can roughly be divided into four parts (Figure 1) – whose boundaries refer to the present day lynx management regions in Norway [31]. The predation study started in the central parts of Hedmark County in 1995, hereafter called Region 5 [10], [35], [36]. In 2001 the study moved south to the area in and around Akershus and Østfold Counties (Region 4). The study in Region 4 ended in 2007. From 2006 to 2011 data were collected in two areas further west, in Buskerud, Telemark, Vestfold and Oppland Counties, hereafter called Region 2 north and Region 2 south [37].

The study area encompasses an environmental gradient as you move from south to north in the area (increased ruggedness and differences in elevation, increasing snow fall, decreasing proportion of farmland and decreasing human density). The north-eastern portion of the study area (Region 5) is characterized by several river valleys at around 200–300 m, separated by hills reaching to 700–800 m. The forest is mainly composed of Norwegian spruce *Picea abies* and Scots pine *Pinus sylvestris* and most of it has been logged and regenerated throughout the last 100 years. The roe deer density in this portion of the study area is generally lower than further south. The south-eastern portion of the study area (Region 4) includes patches of both coniferous and deciduous forest, represented mainly by birch *Betula* spp. and the landscape is more human-modified, with the forest fragmented by cultivated land. The altitude is not higher than 300 m, and roe deer occur at higher densities than further north. The northwestern portion of the study area (Region 2 north) resembles region 5, but with deeper valleys, steeper terrain and higher mountains between the valleys. As we move to the coastal areas in the southwest (Region 2 south), the landscape is more human-modified, and the forest is fragmented by cultivated land. The roe deer density is lower in the northwest compared to further south. This density gradient reflects variation in habitat, snowfall and environmental productivity. Although a few red deer *Cervus elaphus* L. exist in small pockets in Region 5, red deer density is far higher in the two parts of Region 2, which reflects an on-going recolonisation process. Wild mountain reindeer *Rangifer tarandus* were seasonally available at higher altitudes (mainly above treeline) in the two northern parts (Region 2 north and Region 5) of the study area, and moose *Alces alces* occur in high numbers in all areas. Throughout the study area, a wide range of small prey species were available as prey. The most important are mountain hares *Lepus timidus,* red fox *Vulpes vulpes* and forest birds such as black grouse *Tetrao tetrix* and capercaillie *Tetrao urogallus*. We had no available data on small game density in the different areas.

All parts of the study area have free ranging sheep grazing in forest and alpine-tundra habitats from June until September, mainly without effective protection or constraints on their movements. However, the density and distribution of sheep vary considerably inside the area. Lynx depredation focuses almost exclusively on lambs so we expressed all densities as lamb density only. In this study 94% (156) of the sheep found killed by collared lynx where lambs, and found no difference in the proportion of lamb between the four areas (χ2 = 1.8, d.f. = 3, P>0.05). Region 2 north has the widest distribution of grazing areas and the highest densities of sheep, with lamb densities inside each lynx summer home ranging from 0.4 to 6.5 lamb km^−2^. In Region 5 lamb densities range from 0.1 to 10 lamb km^−2^. As we move south (Region 2 south and Region 4), the density of sheep can still be high locally, but sheep grazing areas are smaller and more patchily distributed. Lamb densities in these areas range from 0.2 to 6 sheep per km^−2^.

### Lynx Kill Rates

Lynx kill rates on sheep were sampled during the grazing season (June – September) following different sampling protocols as the telemetry technology developed over the years from 1995 to 2011. Forty-eight individuals (24 males, 24 females) had access to free-ranging sheep inside their summer home ranges, and were included in this analysis. From 1995 to 2003 data on sheep predation were sampled using intensive VHF tracking. Lynx were located every 15 minutes during the night (8 to 12 hours), or during the entire 24 hour period using VHF tracking, and we searched all locations where the collared lynx stopped for at least one hour during its travels at night in order to locate potential kills [35], [38]. From 2004 to 2008, data on sheep predation were sampled using GPS store-on board collars programmed to take 2 to 8 positions per day. Potential predation sites (“clusters”) were identified from GPS-locations after the collar had dropped off the animal using GIS-software (ArcView 3.3, ESRI) and a web-based map-system for displaying telemetry data (http://www.dyreposisjoner.no). The number of locations required to define a cluster, later visited in the field, was at least 2 locations within 100 m. From 2008 to 2011, data on sheep predation were sampled using GPS-GSM collars programmed to take between 12 and 19 GPS-locations a day for 15 to 80 days per summer. The GPS-GSM technology allowed us to visit every cluster (at least 2 locations within 100 m) in the field directly after the lynx had left the area. In addition, all single locations around clusters were visited when logistically possible. For all 3 methods, dogs were often used to search for kills. Because of the store onboard collars (2004–2008), the time elapsed between the lynx had left a kill site and the field visits ranged from 1 to 369 days. However, >80% of the clusters were visited within 20 days after the lynx left the kill site. Lynx rarely scavenge [38]–[40], and 89 out of 185 sheep carcasses found at clusters were defined as probably lynx-killed when we found prey remains (e.g. hair, rumen, bones) that matched the date of the cluster and where there were no other signs of cause of death. The remaining 96 sheep carcasses had clean bite marks to the throat of a sheep when found, and were defined as confirmed lynx-kills.

**Figure 2:**
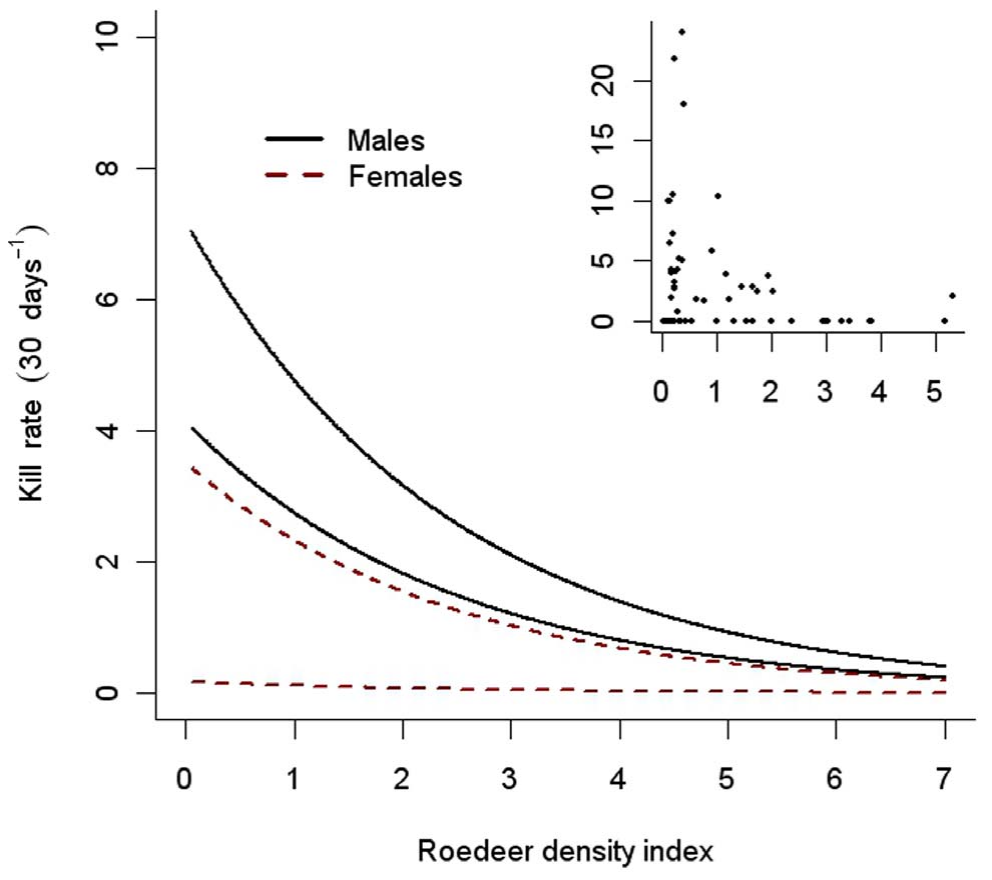
Predicted kill rates (i.e. number of sheep killed in 30 days) under different roe deer densities for male (solid black lines) and female (dashed red lines) lynx. Upper lines for each sex are predicted kill rates under high sheep density (95% percentile of observed lamb densities: 6.6 lamb km^−2^) and lower lines are predictions for low sheep density (5% percentile of observed lamb densities: 0.1 lamb km^−2^). Scatter plot inset in right corner represents the raw data.

Data on lynx kill rates were pooled per lynx per season, and expressed as number of sheep killed in 30 days. Number of days monitored per lynx per season varied from 10 to 122 (mean 39 days SD±31). To scale the kill rate to 30 days we used two procedures. To display raw data (e.g. in Figure 2) we divided number of kills detected by number of days with intensive monitoring, and multiplied by 30. In the zero-inflated models (see *Statistical analyses* under), we used the observed number of kills as the response variable, and the duration of the intensive monitoring as an offset variable (i.e. a factor for which there is an expected slope equal to 1). The lines in Figure 2 are scaled so as to predict 30-day kill rats.

### Roe Deer Density Index

We used 3 year average hunting bag statistics at the municipality level, available from Statistics Norway (www.ssb.no), as an index of local roe deer density. We assume that the spatial variation in our roe deer density index broadly reflects the variations in roe deer density. This assumption has previously been supported by [41] and used in several previous analyses [42], [43]. For ease of interpretation, we rescaled our roe deer density index from number of roe deer shot per forested area to number of roe deer per forested area based on the assumption that the harvesting rate was constant across years and regions (∼15%; based on mortality data from 299 radio-collared roe deer in the study area [44]). For the analysis, the roe deer density index was calculated as the average density across the municipalities in which the individual lynx travelled during summer. Although this density index is crude, we believe that it is functional across such a massive variation in roe deer density (>2 orders of magnitude) and across such a large study area.

### Sheep Density

Spatial data on sheep grazing areas and on the associated area-specific numbers of sheep released in spring were obtained from the Norwegian Forest and Landscape Institute (http://www.skogoglandskap.no/) for 1995–2010. Because lynx depredation on sheep mainly affects lambs (94% of sheep killed in this study were lambs), we only included data on number of lambs available inside each lynx summer home range in our analyses. We used GIS-software (ArcView 3.3, ESRI) to calculate the number of lambs available inside each lynx’s summer home range (June–September). When lynx only utilized parts of a grazing area we assumed that sheep were evenly distributed inside the grazing area, and calculated the number of lambs available to an individual lynx from the proportion of the grazing area that was overlapped by the lynx’s home range.

### Statistical Analyses

Our kill rate data contained many more zeros than expected from a Poisson distribution, thus zero-inflated negative binomial models (ZINB) [45] were used to model the effects of lynx sex, roe deer density and sheep density on observed kill rates on sheep. A ZINB model is a mixture model consisting of two parts; a binomial model (zero-inflation model), with or without covariates used to model the excess zeros, and a count process model including expected zeros modelled with a negative binomial error distribution. Both parts of the process can be modelled as a function of independent variables. ZINB models were appropriate because 1) the existence of excess zeros made a zero-inflated model necessary and 2) excessive variation also after accounting for the zero-inflation made a negative binomial distribution in the count part of the model more appropriate than a Poisson distribution. Although we had observations for 2 seasons for 7 lynx individuals, we did not deal with pseudoreplication [46] by treating individual as a random factor. Primarily this was because there is currently no generally accepted statistical method to deal with autocorrelated error structures in ZINB models [45] and secondly, other sources of spatial and temporal correlation that we could not account for would make the inclusion of individual identity as a random term arbitrary.

We included three independent variables (lynx sex, roe deer density and sheep density). To avoid overfitting (i.e. fitting models with too many parameters with respect to the data), we made two main restrictions: 1) We never fitted models with both roe deer density and sheep density in the same part of the model, and 2) we considered only main effects (although a “biological” interaction might become evident depending on the structure of the two parts of the ZINB model). Even with these restrictions, a total of 36 different models exist, and the most complex models have 7 parameters to be estimated. To ease model selection, we divided the selection process into discrete steps gradually improving the model fit to the data. In the first step, we compared models with lynx sex and roe deer density in both parts of the model and conducted model simplification in the zero-inflation part of the model only. In the second step, we substituted roe deer density with sheep density in zero-inflation model and continued to the procedure of selecting the best predictor variables for the zero-inflation part of the model. Then, in step three we used the “best model” (sensu [47]) from this set of models, but this time we were interested in finding the best predictor variables in the negative binomial part of the model. The performance of the models was investigated based on their AICc values [47], where the model with the lowest AICc value indicates the best model among the examined models, given the data. For each model we also computed Akaike weights (w_i_) [47]. The Akaike weight is a measure of the relative support for each of the models in the subset, given the data and the model subset, and the sum of w_i_ is equal to 1. All models were run in R 2.12.1 [48] with the add on library pscl [49] using the command zeroinfl. Likelihood ratio tests for comparing models were performed with the command lrtest in the add on library lmtest [50]. As the number of tracking days varied between the different study periods, we included log(number of tracking days) as an offset variable (i.e. a variable with a slope equal to one).

## Results

We found 399 prey remains from 48 different lynx monitored during 2161 nights (Table 1). Free-ranging sheep were the most frequently killed prey in the two areas with low roe deer densities (Region 5 and Region 2-north), while roe deer was the most commonly killed prey species in the two areas with high roe deer density (Region 4 and Region 2-south). Twenty-six of the 48 monitored lynx (18 [75%] of the males and 8 [33%] of the females) killed sheep while being monitored. Fifteen (10%) of the 154 killing events on domestic sheep involved multiple killing from 2 to 5 sheep, and all multiple killing were made by males. The highest kill rates on sheep were found in the two areas with the lowest roe deer density and the highest sheep density (Table 2), where male lynx on average killed 8 and 6 sheep per 30 days, respectively.

**Table 1:** Prey found at clusters from Eurasian lynx (*Lynx lynx*) in south-eastern Norway during summer, 1995–2011, grouped by study area (see text for explanation).

	Region 5	Region 4	Region 2 - north	Region 2 – south
	(1995–1999)	(2001–2006)	(2007–2011)	(2008–2011)
	*Low roe / low* *lamb densities*	*High roe / low* *lamb densities*	*Low roe / high* *lamb densities*	*High roe / low* *lamb densities*
Prey species	*n* (%)	*n* (%)	*n* (%)	*n* (%)
Domestic sheep (*Ovis aries*)	32 (32)	10 (25)	117 (62)	16 (23)
Domestic goat (*Capra hircus*)	3 (3)			
Roe deer (*Capreolus capreolus*)	22 (22)	24 (60)	19 (10)	36 (52)
Red deer (*Cervus elaphus*)			1 (0.5)	5 (7)
Reindeer (*Rangifer tarandus*)	4 (4)		1 (0.5)	
Moose (*Alces alces*)^a^		1 (3)	1 (0.5)	
Mountain Hare (*Lepus timidus*)	16 (16)	1 (3)	28 (15)	5 (7)
Capercaillie (*Tetrao urogallus*)	12 (12)		9 (5)	4 (6)
Black grouse (*Tetrao tetrix*)	4 (4)	1 (3)	5 (3)	1 (2)
Red fox (*Vulpes vulpes*)		1 (3)	1 (0.5)	
Other mammals^b^	5 (5)			1 (2)
Other birds^c^	3 (3)		6 (3)	1 (2)
Scavenging events^d^		2 (5)	1 (0.5)	
Total prey	101	40	189	69
Lynx individuals	18	7	14	9
Monitoring days	483	446	854	378

Percentages are based on frequency of occurrence.

a Calves.

b Lemming (*Lemmus lemmus*), Brown rat (*Rattus norvegicus*), 3 red squirrels (*Sciurus vulgaris*), and 1 Eurasian beaver (*Castor fiber*).

c 3 Wood Pigeon (*Columba palumbus*), 1 Meadow Pipit (*Anthus pratensis*), 6 unknown birds.

d 2 moose carcasses, 1 roe deer carcass.

**Table 2:** Average number of sheep killed per 30 days, estimated lamb and roe deer densities, in four study areas in southern Norway during summer, 1995–2011.

Area	Sex	Lynx*season	Proportion (%) of lynx involvedin depredation (n)	Average days	Average lamb per km^2^	Average roe per km^2^	Sheep killed per 30 days
Region 5	M	9	83% (6)	17 (±7)	1.3 (±2.6)	0.2 (±0.2)	7.9 (±8.6)
	F	14	8% (12)	24 (±14)	1.0 (±1.1)	1.1 (±1.3)	0.2 (±0.7)
Region 4	M	5	25% (4)	76 (±46)	1.8 (±2.5)	3.5 (±1.8)	0.4 (±1.8)
	F	3	33% (3)	23 (±12)	1.9 (±2.4)	2.2 (±1.1)	0.8 (±1.4)
Region 2 - north	M	8	100% (8)	63 (±34)	3.2 (±1.8)	0.6 (±0.4)	5.9 (±3.1)
	F	7	83% (6)	50 (±34)	5.2 (±3.0)	0.4 (±0.3)	2.4 (±1.8)
Region 2 - south	M	6	67% (6)	34 (±12)	1.1 (±0.7)	3.2 (±1.4)	1.9 (±1.6)
	F	3	33% (3)	59 (±19)	1.5 (±0.9)	2.7 (±2.1)	0.9 (±1.6)

Standard deviations in brackets.

The ZINB model including the additive effects of lynx sex and sheep density in the zero-inflation model, and the additive effect of lynx sex and roe deer density in the negative binomial part of the model received most support (Table 3; Figure 2). The second best ranked model included the additive effects of lynx sex and sheep density in the zero-inflation model and the effect of roe deer density in the negative binomial part. This model was 4.5 times less likely to be the best model based on the Akaike’s weights (w_i_). In addition, a likelihood-ratio test clearly suggested that the effect of roe deer density was needed to adequately describe the data (χ^2^ = 5.651, df = 1, p = 0.017). Consequently, the best supported model suggested that the number of free-ranging sheep killed by an individual lynx in southern Norway is best understood as a combination of the individual effects of sheep density, roe deer density, and lynx sex (Table 4, Figure 2). Highest kill rates were found for males in areas with high sheep densities and low roe deer densities. Irrespective of sheep density and sex, the lowest kill rates occurred in areas with high density of roe deer. As roe deer density decreased, males killed sheep at higher rates, and this pattern holds for both high and low sheep densities. Similarly, females killed sheep at higher rates in areas with high densities of sheep and low densities of roe deer. In areas with low sheep densities, our model suggests that female lynx rarely kill free ranging sheep even when densities of their main wild prey (i.e. roe deer) are low.

**Table 3:** AICc values for all evaluated models, together with ΔAIC values.

	Model Specification	Df	AICc	dAICc	wi
Step I	Roe+Sex|Roe+Sex	7	214.0006	22.936	<0.001
	Roe+Sex|sex	6	216.4416	25.377	<0.001
	Roe+Sex|Roe	6	216.2099	25.145	<0.001
	Roe+Sex|1	5	217.1654	26.101	<0.001
Step II	Roe+Sex|Lam+Sex	7	191.0649	0.000	0.746
	Roe+Sex|Lam	6	202.6714	11.607	0.002
Step III	Sex|Lam+Sex	6	196.1979	5.133	0.057
	Roe|Lam+Sex	6	194.0824	3.018	0.165
	Lam+Sex|Lam+Sex	7	198.7967	7.732	0.016
	Lam|Lam+Sex	6	201.2709	10.206	0.005
	1|Lam+Sex	5	199.7381	8.673	0.010

The models are presented in the format of the R-language, so that x_1_+x_2_|x_3_+x_4_ is read so that the first part represents the negative binomial part of the model (x_1_+x_2_;”count process”) whereas the latter part is the referring to the zero-inflated part of the model (x_3_+x_4_ ;excess zeros). [lam = lamb density, sex = lynx sex, roe = roe deer density index].

**Table 4:** Parameter estimates for the best model in the set of candidate models (presented in Table 3).

Model term	Parameter	Se	z-score	P-value
*Negative* *Binomial model*				
Intercept	−1.462	0.180	−8.119	<0.001
Sex [Females]	−0.705	0.282	−2.502	0.012
Roe	−0.356	0.130	−2.746	0.006
*Zero-inflation* *model*				
intercept	0.673	0.909	0.740	0.459
Sex [Females]	5.473	3.519	1.555	0.120
Lam	−2.962	2.076	−1.427	0.154

Parameters are presented on the link-scale (log-link in the negative binomial model; logit-link in the zero-inflation model).

* log(*theta)* parameter of the negative binomial model estimated at 1.498 (se = 0.490).

## Discussion

This study demonstrates how social and ecological factors interact to influence a predator’s depredation rate on livestock. We found that variation in sheep density, roe deer density, and lynx sex are all needed to understand the variation in lynx depredation rates on free-ranging sheep in southern Norway. The direction of the results, that sheep depredation is inversely related to wild prey density, are in agreement with the predictions from the *energetic model*, with the highest kill rates on sheep in areas with low densities of their main prey, roe deer. This result does not necessarily contradict predictions from the *attraction model* which we have found support for in an early analysis [10]. The difference may simply be a matter of scale. As demonstrated here, and in several other studies, the depredation pressure on livestock can be higher when the overall wild prey densities are less abundant [6], [15], [16], [18], [22], [51], [52]. On a fine scale (within sections of a home range) depredation may be linked to patches of high ungulate densities, because carnivores may just spend more time in the most prey rich patches, leading to higher encounter rates with livestock and therefore more incidental depredation [8]–[10], [53].

When we first started to study lynx depredation on sheep in Region 5 (1995–1999) we concluded that lynx depredation on sheep seemed to be more due to chance encounters between lynx and sheep, rather than the results of lynx actively seeking sheep as prey [10], [35], [36]. In this area sheep densities were greater than roe deer densities within most of the monitored lynx home ranges. Still sheep only constituted about 26% of the digestible biomass in lynx diet during summer estimated from the frequency of lynx kills and the relative consumption of each kill [10]. Here, the avoidance of abundant free-ranging sheep seemed to reflect some intrinsic aversion to sheep [35] indicating that depredation on livestock and predation on wild prey are based on different processes. However, when adding another 10 years of data from an expanded study area, the data suggest that lynx can switch from roe deer, to sheep depending on their relative abundance. In a multiple prey system, an opportunistic predator should respond to variations in prey densities by changing its relative use of prey [54]. Decreasing densities of prey are likely to result in decreased encounter rates and thus increased searching time. The diet shift observed in our study might be explained by changes in lynx-sheep encounter rates with changes in the relative abundance of roe deer and sheep. In our study, the estimated average lamb: roe deer density ratio inside lynx summer home ranges varied from 0.6∶1 in the south to 4∶1 in the northwest, and the northwestern area was the only area were sheep dominated the diet for both sexes.

In all our study areas male lynx killed sheep more frequently than females, given the same ecological settings. There is little comparable data on systematically sampled kill rates on livestock from a substantial sample of individual predators, but a few other studies of depredation by carnivores including leopard *Panthera pardus*, cougar *Puma concolor*, and black bear *Ursus americanus*, support this pattern of males killing more livestock than females [55]–[57]. The best comparable data comes from studies on coyotes *Canis latrans*, which have shown that most depredation is due to breeding adults and especially males [13], [58], [59]. However, telemetry studies of lynx in France, jaguars *Panthera onca* and grizzly bears *Ursus arctos* did not find any apparent effect of sex [7], [60], [61] on predation. The fact that male lynx killed sheep more frequently than females can partly be explained by the larger home ranges of males compared to females [42], [62]. Males move faster and over larger areas, and thereby encounter sheep more often than females, but perhaps the most important sex difference is that males were responsible for all recorded cases of multiple killing in our study. Multiple killing of livestock is quite common among carnivores [4], and is typically associated with unusual environmental conditions that restrict the prey’s ability to escape [63]. When faced with an abundant, vulnerable prey like sheep, there does not seem to be an adaptive reason why a lynx should limit killing, as the threat of injury is low [4]. The extent of multiple killing was disproportional to the greater energetic needs of males, and both sexes of lynx would tend to encounter sheep in the same setting, therefore these results probably reflect some intrinsic aspects of male behaviour, akin to their greater willingness to take risks [64].

## Conclusions

This study confirmed our earlier findings [35] that a high proportion of lynx, especially males, will kill sheep at some stage as long as unguarded sheep are found at high densities throughout the natural habitats exploited by lynx. When depredation is not due to a few specific problem individuals, selective removal is impossible and lethal control will only reduce depredation if it reduces the overall number of lynx in the population [65]. Comparative studies of lynx from France and Sweden have shown that confining sheep in fenced fields or on alpine pastures (out of the forest) dramatically reduces depredation losses per lynx [7], [53], [66].

The present day Norwegian *ex post facto* compensation system creates widespread social conflicts because only a small fraction of the compensated sheep losses are documented through a formal examination of a carcass [38], [67]. Combined with accurate lynx monitoring data, our findings on how lynx depredation rates vary with different factors can be used to evaluate current compensation levels based on empirical data, instead of a qualified guess of estimated losses by regional managers as it is today. This may be the first step towards a compensation system based on objective, accurate, and area specific estimation of sheep losses likely to be due to lynx. This would also facilitate transition to a risk based incentive system [26], [68] as such systems are believed to encourage depredation prevention rather than damage documentation [69].
